# *Streptococcus suis* Serotype 2 Biofilms Inhibit the Formation of Neutrophil Extracellular Traps

**DOI:** 10.3389/fcimb.2017.00086

**Published:** 2017-03-20

**Authors:** Fang Ma, Li Yi, Ningwei Yu, Guangyu Wang, Zhe Ma, Huixing Lin, Hongjie Fan

**Affiliations:** ^1^College of Veterinary Medicine, Nanjing Agricultural UniversityNanjing, China; ^2^College of Life Science, Luoyang Normal UniversityLuoyang, China; ^3^National Center of Meat Quality and Safety Control, Nanjing Agriculture UniversityNanjing, China; ^4^Jiangsu Co-Innovation Center for Prevention and Control of Important Animal Infectious Diseases and ZoonosesYangzhou, China

**Keywords:** zoonosis, streptococcus toxic shock-like syndrome, NETs, bacterial biofilm, clinical therapy

## Abstract

Invasive infections caused by *Streptococcus suis* serotype 2 (SS2) has emerged as a clinical problem in recent years. Neutrophil extracellular traps (NETs) are an important mechanism for the trapping and killing of pathogens that are resistant to phagocytosis. Biofilm formation can protect bacteria from being killed by phagocytes. Until now, there have only been a few studies that focused on the interactions between bacterial biofilms and NETs. SS2 in both a biofilm state and a planktonic cell state were incubated with phagocytes and NETs, and bacterial survival was assessed. DNase I and cytochalasin B were used to degrade NET DNA or suppress phagocytosis, respectively. Extracellular DNA was stained with impermeable fluorescent dye to quantify NET formation. Biofilm formation increased up to 6-fold in the presence of neutrophils, and biofilms were identified in murine tissue. Both planktonic and biofilm cells induced neutrophils chemotaxis to the infection site, with neutrophils increasing by 85.1 and 73.8%, respectively. The bacteria in biofilms were not phagocytized. The bactericidal efficacy of NETs on the biofilms and planktonic cells were equal; however, the biofilm extracellular matrix can inhibit NET release. Although biofilms inhibit NETs release, NETs appear to be an important mechanism to eliminate SS2 biofilms. This knowledge advances the understanding of biofilms and may aid in the development of treatments for persistent infections with a biofilm component.

## Introduction

*Streptococcus suis* (SS) is a major swine pathogen that causes a variety of diseases such as septicemia, meningitis, and endocarditis, which lead to economic losses. *S. suis* serotype 2 (SS2) is considered the most pathogenic and prevalent capsular type (Wertheim et al., 2009; Kerdsin et al., 2016). People working with pigs or people who consume pork-derived products from infected animals are at risk. During the last decade, several human epidemic outbreaks were reported in Asia and all over the world (Gottschalk et al., 2010a,b; Goyette-Desjardins et al., 2014). In addition, streptococcus toxic shock-like syndrome (STSLS), a peracute infection characterized by shock and a high mortality rate, is reported to be caused by SS2, resulting in increased public health concerns worldwide (Tang et al., 2006; Gomez et al., 2014).

Bacterial biofilms are bacterial communities and are an important mechanism for bacterial resistance to immune system pressures and antimicrobials (Bojarska et al., 2016). Most of SS2 clinical isolates can form biofilms, which contribute to persistent infection, transmission and difficulties to eradicate infection (Bojarska et al., 2016). However, little information is available on the interaction between the host immune system and SS2 biofilms (Thurlow et al., 2011; Yang et al., 2016). Neutrophil extracellular traps (NETs), which are composed of granule and nuclear constituents, are made by activated neutrophils (Brinkmann et al., 2004; Uhlmann et al., 2016). The nuclear constituents are DNA and histones; DNA is the backbone of NETs and traps the pathogens by charge interactions (Wartha et al., 2007). In the recent years, NETs have been identified as a significant antibacterial mechanism employed by neutrophils (Csomos et al., 2016). Neutrophils are observed to generate NETs upon activation with interleukin-8 (IL-8), phorbol myristate acetate (PMA), lipopolysaccharide (LPS), and various microbes (Leshner et al., 2012). NETs can disarm and kill a variety of pathogens, including GAS, *S. aureus, Shigella flexneri*, and fungi, by capturing the microbes and providing a high local concentration of antimicrobial granules (Brinkmann et al., 2004; Buchanan et al., 2006; May et al., 2015). NETs have been found to be abundant at *in vivo* sites of infection and inflammation, including in cases of the autoimmune disease systemic lupus erythematosus and a murine model of pneumococcal pneumonia (Beiter et al., 2006; Hakkim et al., 2010).

In previous studies examining the immune system response to various microorganisms, certain microbes have been shown to evade phagocytosis but become entrapped by NETs (Branzk et al., 2014). *Candida albicans* biofilms evade phagocytosis and impair NET formation (Johnson et al., 2016). However, the nature of bacteria and fungi is dramatically different, particularly in size and biofilm structure. Therefore, whether bacterial biofilms can stimulate NET formation is unknown and the influence of SS2 biofilms on bacterial survival in NETs is unclear and requires exploration.

Bacterial biofilm formation allows bacteria to persist in the host, making the treatment of streptococcosis challenging (Walker et al., 2005). Cases of human infections worldwide stress the lack of knowledge on the virulence and interactions with host immune cells. Our study provides further knowledge on SS2 biofilm and immune response interactions, which can lead to novel approaches to streptococcosis clinical therapy. Further understanding of host-SS2 interactions may help to explain the complex evolution of the emerging human threat.

## Materials and methods

### Ethics statement

This study was carried out in an accordance to animal welfare standards and were approved by the Ethical Committee for Animal Experiments of Nanjing Agricultural University, China. All animal experiments accorded with the guidelines of the Animal Welfare Council of China.

### Bacterial strains and cells

The wild-type SS2 strain ZY05719 is an isolate from Jiangsu Province and was grown in Todd-Hewitt broth (THB) medium (Difco, BD, Franklin, NJ, USA) at 37°C on a gently rocking shaker. The bacteria were cultured to the mid-exponential phase and were collected in media for the experiment using planktonic cells. SS2 biofilms were identified with Congo Red Agar composed of 3% THB, 0.08% Congo Red (Sigma Aldrich, St. Louis, Mo, USA), 0.5% glucose (Biosharp, Anhui, China) and 1.5% agar powder. Neutrophils from the bones of mice were cultured in RPMI 1640 (Gibco-BRL, Thermo Fisher Scientific, Waltham, MA, USA) supplemented with 2% heat-inactivated fetal bovine serum (FBS) at 37°C in 5% CO_2_. RAW264.7 cells (ATCC® TIB-71™) were purchased from the American Type Culture Collection (ATCC) and were cultured in Dulbecco's modified Eagle's medium (DMEM; Wisent, Canada) supplemented with 10% FBS at 37°C in 5% CO_2_.

### Isolation of neutrophils from mouse bone marrow

Neutrophils were isolated from 4-week-old ICR mice as previously described with modifications (Zhao et al., 2015). Briefly, the mice were euthanized and sprayed with 70% ethanol. The bone marrow from the tibias and femurs was flushed with sterile PBS with a 20-gauge needle into a 15 ml Falcon tube (BD Falcon), and the cells were washed by centrifugation at 400 × g for 10 min. The cell pellet was resuspended in 3 ml of PBS. A Percoll (Sigma Aldrich) density gradient was prepared in a 15 ml Falcon tube by the careful addition of 3 ml of 80% Percoll followed by 3 ml of 65% Percoll and 3 ml of 55% Percoll. The cell suspension was overlaid carefully and centrifuged for 30 min at 1000 × g at room temperature. The top layer and the 55% Percoll layer were carefully aspirated and discarded. The cells at the 80/65% gradient interface were collected and then washed and suspended in RPMI1640 medium. A greater than 90% neutrophil purity was confirmed by Trypan blue staining and flow cytometry.

### Neutrophil detection by flow cytometry

Flow cytometry was performed as previously described with modification (Barletta et al., 2012). The neutrophils were stained with 0.1 μg of FITC-mouse Ly6G antibody (eBioscience, San Diego, CA, USA) and 0.1 μg of PE-mouse CD11b antibody (eBioscience). All of the experiments were recorded using Accuri Cflow software (BD Bioscience, CA, USA) and were analyzed using FlowJo software (Three Star, Ashland, OR, USA).

### Biofilm formation *in vitro*

SS2 biofilm formation required the presence of fibrinogen in the culture medium (Freeman et al., 1989; Bojarska et al., 2016). For SS2 biofilm formation *in vitro*, 100 μl of THB with 2.5 mg/ml of human plasma fibrinogen (Sigma Aldrich) and 100 μl of the bacterial suspension at a concentration of 10^6^ colony forming units (CFU)/ml were incubated in a 96-well plate at 37°C for 24 h. Each well was washed carefully with PBS to remove planktonic bacteria. For biofilm experiments, the biofilms were resuspended in PBS by repeated pipetting, through which the biofilms were physically dispersed (Johnson et al., 2016). To detect the effect of neutrophils on SS2 biofilm formation, 100 μl of a 10^4^ CFU/ml bacteria suspension in RPMI was mixed with 100 μl of purified neutrophils at 10^6^/ml with or without DNase I or cytochalasin B in a 96-well plate and incubated for 24 h at 37°C in 5% CO_2_. The purified neutrophils without bacteria were included as a negative control. DNase I and cytochalasin B were added to bacterial suspension only in the presence of fibrinogen to evaluate the influence of these two inhibitors on biofilm formation.

Biofilm formation in the above assay was detected in a 96-well plate using a 0.1% crystal violet stain (Kosikowska et al., 2016). After incubation for 24 h, the plates were washed three times with PBS to remove nonadherent cells. To each well, 200 μl of methyl alcohol was added to fix the cells, and then the plates were placed in a 37°C dryer oven to remove the methyl alcohol. After the plates were washed with PBS three times, the biofilm in each well was stained with 200 μl of 0.1% crystal violet for 20 min. Following staining, the plates were washed three times, and the crystal violet staining the cells was dissolved with 95% ethyl alcohol. The biofilm was detected with a multifunctional microplate reader (Tecan Infinite Pro, Austria) at an optical density (OD) of 595 nm.

### Biofilm detection *in vivo*

To determine whether SS2 forms biofilms *in vivo*, the bacteria were grown to an OD_600_ of 0.8 and were then washed three times with PBS. Four-week-old ICR mice were challenged with SS2 ZY05719 at 10^8^ CFU/ml by intraperitoneal injection. Three days post-injection, the mice were challenged again. At 12 h after the injection, the mice were euthanized, and the heart, liver, spleen, lungs, and kidneys were collected and homogenized. SS2 biofilm formation was determined using plate streaking in modified Congo Red THB plate agar. In order to exclude the color change of Congo Red THB plate is caused by planktonic bacteria or tissue homogenate, planktonic ZY05719 was added into organs homogenate of non-injected mice and the mixture was streaked on Congo Red THB plate directly. The bacteria isolated *in vivo* were detected with polymerase chain reaction (PCR) using the primer combinations GAPDHF/GAPDGR to detect SS2 GAPDH: forward, 5′-CATGGACAGATAAAGATGG-3′; reverse, 5′-GCAGCGTATTCTGTCAAACG-3′ and CPSF/CPSR to detect the SS2 serotype: forward, 5′-GACGGCAACATTGTTGAGTC-3′; reverse, 5′-CTCCTAACCACTGTTCAGTG-3′.

### Phagocytosis assay

The phagocytosis assay was performed with RAW264.7 cells as previously described with some modifications (Mitterstiller et al., 2016). Briefly, RAW264.7 cells were incubated in 24-well plates, and then the cell monolayers were washed three times with PBS. An aliquot (100 μl) of suspension containing 10^6^ CFU/ml planktonic cells or biofilm cells were added to the cells. The 24-well plate was centrifuged at 800 × g for 10 min and was incubated for 2 h at 37°C in 5% CO_2_. Next, the cells were washed with DMEM and were treated with 200 μg/ml of penicillin-streptomycin for 1 h to kill extracellular bacteria. The cells were washed with DMEM, and then 100 μl of trypsin and 900 μl of sterile deionized water were added per well to release the bacteria. The viable bacteria number was determined by plating serial dilutions. Bacteria incubated in DMEM for 2 h without RAW264.7 cells were served as control group to quantify the initial inoculum. The level of phagocytosis was calculated as (CFUs of viable bacteria in experimental group)/(CFUs of viable bacteria in the control group).

### Neutrophil and NET bactericidal assays

The neutrophils bactericidal assay was performed according to a previous method with slight modifications (Uchiyama et al., 2015). The neutrophils were divided into 3 groups: an untreated group containing only purified neutrophils, and two groups of neutrophils were treated with either DNase I (Sigma Aldrich) to inhibit NET formation or with cytochalasin B (Sigma Aldrich) to suppress neutrophil phagocytosis. Bacteria at 3 × 10^7^ CFU were added to the neutrophils at a multiplicity of infection (MOI) of 10. After incubation with planktonic SS2 or biofilm cells for 90 min, the neutrophils were permeabilized with 0.2% Triton X-100 (Sigma Aldrich) on ice to release the intracellular bacteria. The surviving bacteria were diluted and plated on THB agar, and the CFUs were counted. Bacteria without incubation were serially diluted and plated to quantify the initial inoculum.

The neutrophils were stimulated by PMA (200 nM, Sigma Aldrich) for 4 h to form NETs as previously described (Ma et al., 2017). Thereafter, the mixtures were centrifuged at 800 × g for 10 min to remove the cells. Planktonic SS2 and biofilm cells at 2 × 10^7^ CFU were added to the NET supernatant and were incubated for 1 h at 37°C in 5% CO_2_. The bacteria without incubation in NETs supernatant were diluted and plated on THB agar as a control.

### Bacterial survival in mouse blood *in vivo*

Bacterial survival in the blood was determined as previously described (Derkaoui et al., 2016). An aliquot (200 μl) of planktonic or biofilm SS2 at an OD_600_ of 0.5 was injected into mice via the tail vein route. To further evaluate the function of NETs, the bacteria were injected into the tail vein with DNase I (10 mg/kg of body weight), and at 12 h post-infection, DNase I was injected again. Planktonic and biofilm SS2 cells without incubation were plated on THB agar as a control. At 2, 4, 8, and 24 h post-infection, blood was collected via heart puncture, and the blood was serially diluted and plated on THB agar plates.

### Visualization and quantification of NETs *in vitro*

NETs were observed *in vitro* as previously described (Yost et al., 2016). Briefly, neutrophils were pretreated with cytochalasin B for 15 min before incubation with PMA and bacteria. Planktonic ZY05719 at an OD_600_ of 0.6–0.8 were washed twice with PBS, added to the neutrophils at an MOI of 10 on poly-L-lysine-coated cover slides, and then centrifuged at 800 × g for 10 min. After incubation at 37°C for 3 h, the cover slides were fixed with 4% paraformaldehyde for 10 min, permeabilized with 0.1% Triton X-100 and were then blocked with donkey serum at 4°C overnight. The samples were stained with the primary rabbit anti-neutrophil histone H4 antibody (citrulline 3, 1:1000 diluted, Merck Millipore, Billerica, MA, USA) for 1 h at RT, followed by incubation with goat anti-rabbit Alexa 568 antibody (1:100 dilution, Jackson ImmunoResearch, West Grove, PA, USA). The DNA was visualized by staining with 4',6-diamidino-2-phenylindole (DAPI, Thermo Fisher). The images were recorded using a fluorescence microscope (Zeiss, Germany). Bacteria entrapped by NETs were stained with SYTO 9 green fluorescent nucleic stain (Thermo Fisher) and observed using 100 × oil objective.

The NET quantification assay was performed as previously described (Riyapa et al., 2012). For determining the capacity of neutrophils to form NETs in the presence of planktonic and biofilm SS2, 200 μl of neutrophils were incubated with 20 μl of planktonic SS2 cells, biofilm cells or bacteria separated from biofilm matrix with or without PMA for 3 h. The biofilms were disrupted and the mixture was centrifuged at 3,000 × *g* for 10 min to separate the bacteria from biofilm matrix. The supernatant was biofilm extracellular matrix and was collected. The precipitate was bacteria that were separated from matrix and the bacteria were washed 3 times with PBS. As a positive control, neutrophils were stimulated with 200 nM PMA. The corresponding bacteria or biofilm matrix were incubated in media without neutrophils to eliminate the background fluorescence. The negative control was the purified neutrophils incubated in media. Extracellular DNA was used to evaluate the quantity of NETs, which was quantified using a Quant-iT Picogreen dsDNA assay kit (Invitrogen). Briefly, after incubation, the reaction mixture were centrifuged at 800 × g for 5 min to discard the cells. Subsequently, 100 μl of supernatant was added to 100 μl of a working solution, which was then mixed thoroughly. After incubation for 5 min, the fluorescence was read with a multifunctional microplate reader (Tecan Infinite Pro) at 480 nm (excitation)/520 nm (emission).

### Statistical analysis

All experiments were repeated at least 3 times. Student's *t*-test and the GraphPad Prism 5 Software package (GraphPad Software, La Jolla, CA, USA) were used to perform statistical analyses. Values of *p* < 0.05 were considered statistically significant.

## Results

### SS2 biofilm formation improved in the presence of neutrophils

**Figure 1:**
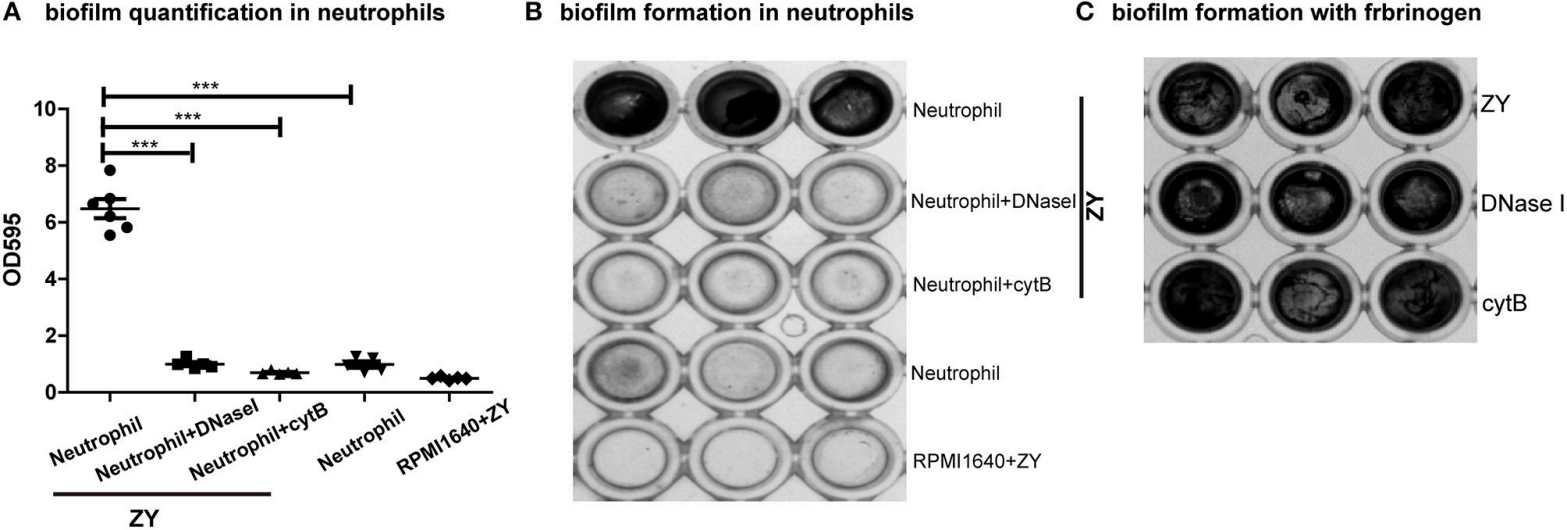
**Neutrophil impact on SS2 biofilm formation ***in vitro*****. SS2 ZY05719 (referred as ZY in figures) biofilm quantification using crystal violet staining. **(A)** The biofilms were quantified using a multifunctional microplate reader at OD 595. When DNase I and cytochalasin B (referred as cytB in figures) were added, the biofilm formation capability decreased up to five-fold. The results are depicted as the mean ± SD (*n* = 5). ^***^p < 0.001. **(B)** The corresponding photos of biofilms stained with crystal violet are shown. **(C)** The influence of DNase I and cytochalasin B on SS2 biofilm formation in the presence of fibrinogen. These two inhibitors have no effect on SS2 biofilm formation.

Wild-type SS2 can form biofilms only in the presence of fibrinogen *in vitro*; however, we found that wild-type SS2 can form biofilms without fibrinogen in the presence of neutrophils. Either phagocytosis or NETs of neutrophil is inhibited, the formation of SS2 biofilm decreased (Figures 1A,B). DNase I and cytochalasin B were used to degrade NET DNA and inhibit phagocytosis and these two inhibitors had no influence on SS2 biofilm formation in the presence of fibrinogen (Figure 1C). This results suggest that in the presence of neutrophil infiltration, SS2 is more liable to form biofilms, which may enhance bacterial survival.

### Identification of SS2 biofilms in mouse organs

**Figure 2:**
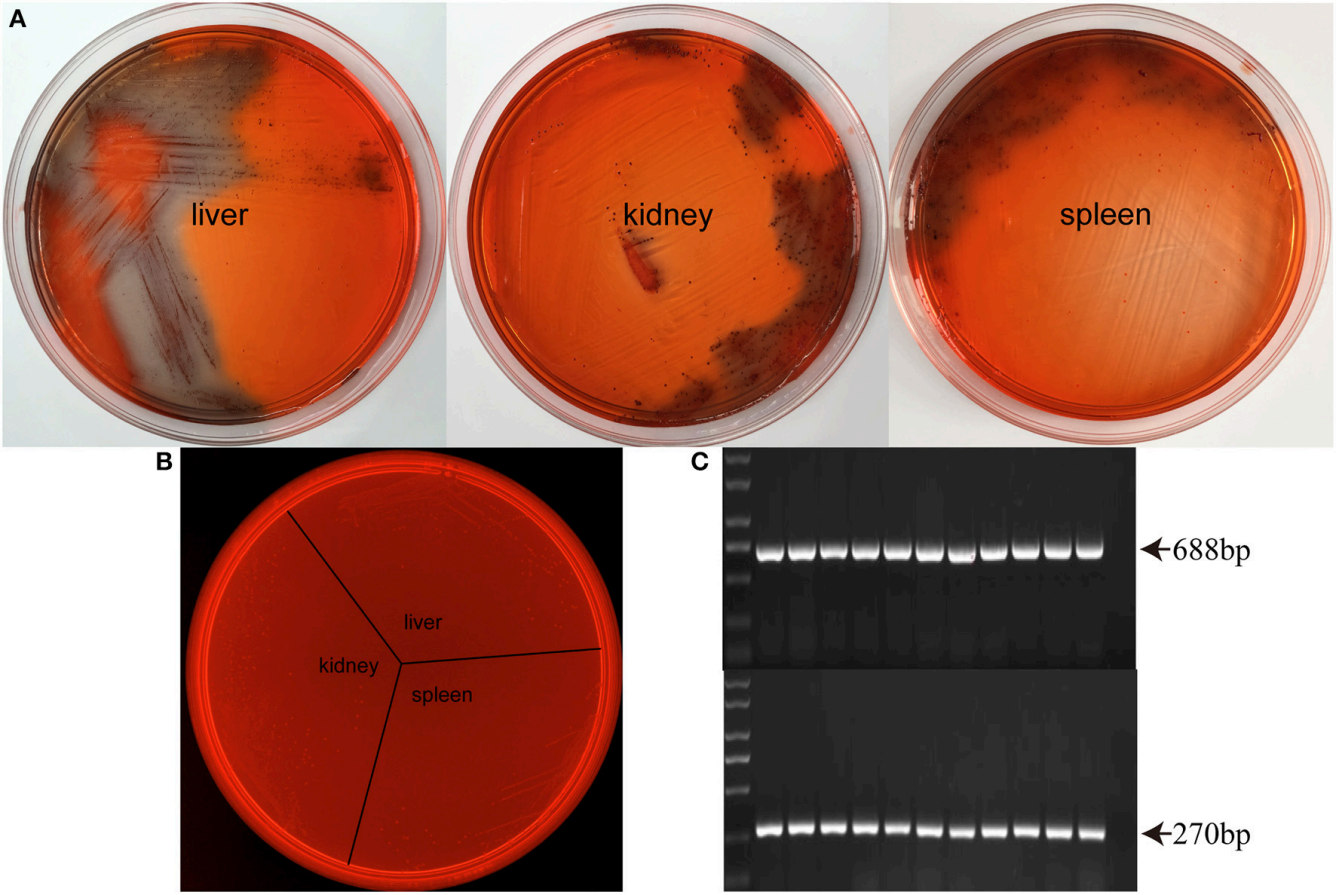
**Biofilm formation in SS2 infected mice. (A)** At 12 h after the second injection with SS2 ZY05719, the mouse organs were homogenized, and the bacterial colonies were examined on modified Congo Red THB agar. Biofilm cells isolated from the liver, kidney and spleen can cause a red to black color change. **(B)** Organs collected from healthy mice were homogenized and mixed with planktonic SS2 ZY05719. The mixtures were streaked on Congo Red THB agar. Planktonic bacteria, tissue homogenate and their simple mixtures cannot cause a color change. **(C)** Isolated bacteria were confirmed as SS2 by PCR. A PCR product of 688-bp indicated the presence of SS2 GAPDH, and the 270-bp product indicated the presence of the SS2 capsule.

Considering that SS2 biofilm formation in THB requires fibrinogen, it is necessary to determine whether SS2 forms biofilms *in vivo*. Biofilm formation can cause a color change of the Congo Red Agar from red to black. Importantly, biofilm SS2 was isolated from the liver, spleen, and kidney of healthy mice challenged with planktonic SS2 (Figure 2A). Control groups are designed to evaluate the influence of planktonic bacteria and tissue homogenate on Congo Red Agar, and the results showed that normal tissue homogenate, planktonic SS2 and even their simple mixtures cannot cause a color change of the Congo Red Agar (Figure 2B). The isolated biofilm cells were confirmed by PCR (Figure 2C). These results indicated that SS2 could form bacterial biofilms during the process of infection.

### Chemotaxis of neutrophils to the site of infection site with planktonic and biofilm cells

**Figure 3:**
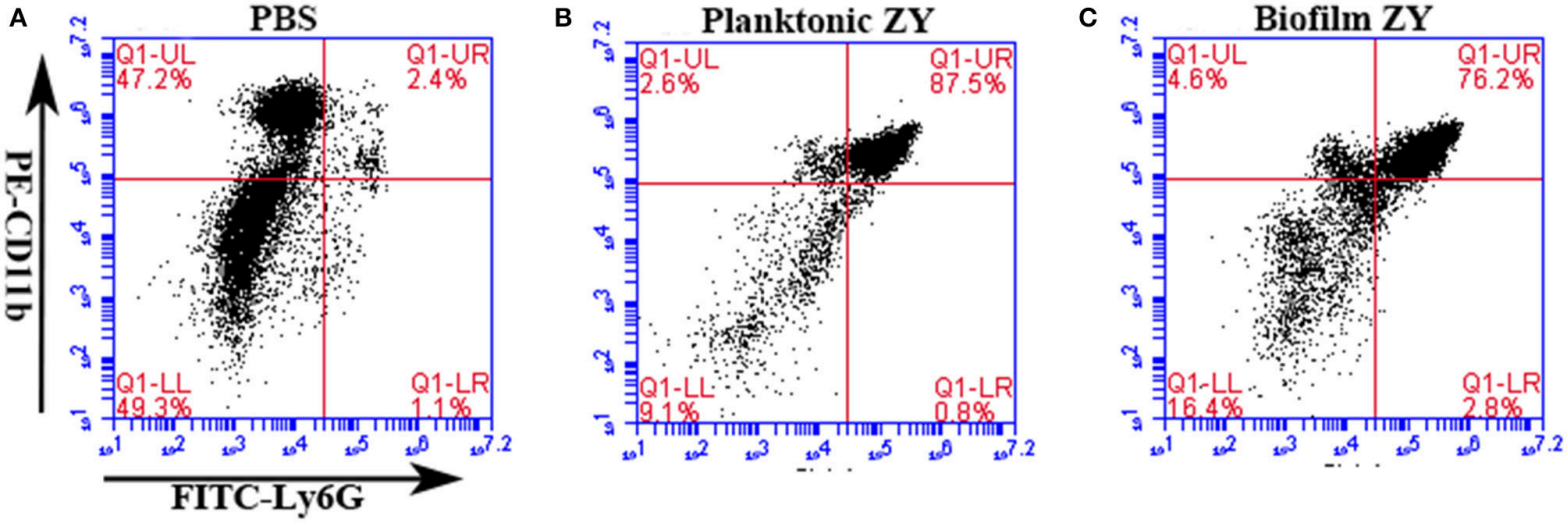
**Proportion of neutrophils in peritoneal washes induced by planktonic and biofilm SS2**. Neutrophils were marked with PE-conjugated anti-CD11b antibody and FITC-conjugated anti-Ly6G antibody. **(A)** Neutrophils comprised 2.4% of the cells collected from mice injected with the PBS control. **(B,C)** Neutrophils comprised 87.5 and 76.2% of cells collected from mice infected with planktonic and biofilm SS2, respectively.

The mice were infected with biofilms and planktonic SS2 at the same OD_600_ using the murine peritoneal infection model; then, mouse immune cells were collected from peritoneal lavage fluids. Cells collected from mice without infection were served as blank control (Figure 3A). The results indicated that both planktonic and biofilm cells caused a significant increase in neutrophils in the peritoneal cavity, from 2.4 to 87.5% and 76.2%, respectively (Figures 3B,C). The neutrophil infiltration provides an environment for the interaction between neutrophils and pathogens.

### Phagocytosis efficiency of biofilm cells and planktonic cells

**Figure 4:**
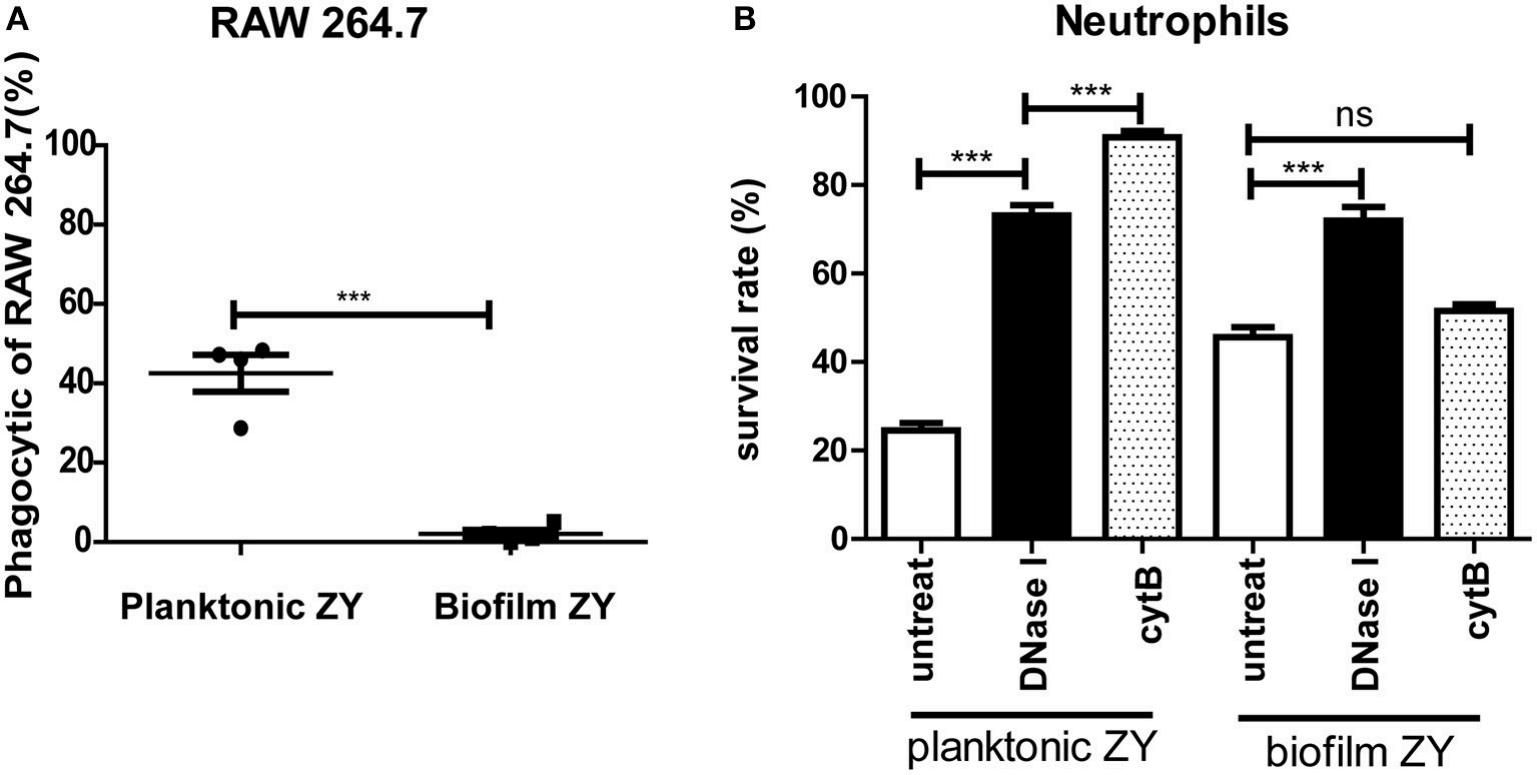
**Phagocytosis by RAW264.7 cells and bactericidal activity of neutrophils. (A)** Planktonic and biofilm SS2 cells were phagocytized by RAW264.7, and 40% of planktonic SS2 cells were phagocytized; however, few biofilm SS2 cells were phagocytized. **(B)** Planktonic and biofilm SS2 cells were killed by neutrophils, and the survival rate of biofilm cell was almost twice that of planktonic cells. When neutrophils were treated with DNase I and cytochalasin B, the survival rate of planktonic cells were increased by 2-fold and 3-fold, respectively. When neutrophils were treated with DNase I, the viable biofilm cells increased significantly; however, when neutrophils were pretreated with cytochalasin B, there was no significant difference between the untreated control and pretreatment groups. The results are depicted as the mean ± SD (*n* = 5). ^***^p < 0.001; ns, no difference between the groups.

The results of the phagocytosis assay indicated that approximately 40% of planktonic SS2 can be engulfed by RAW246.7 cells, a type of professional phagocyte. However, only a few biofilm cells could be plated on THB agar, which indicated that it was more difficult to engulf biofilm SS2 (Figure 4A). Purified neutrophils had a significant bactericidal effect, and the survival capability of biofilm SS2 was nearly twice that of planktonic SS2. When DNase I was added with neutrophil, the survival rate of planktonic bacteria were nearly 2 times greater than that of the corresponding untreated group. The bacterial survival rate in the biofilm group with DNase I treatment was nearly 30% higher than that of the corresponding untreated control group. For planktonic SS2, when neutrophil phagocytosis was suppressed, the survival rate of planktonic SS2 was increased significantly. However, regardless of phagocytosis inhibition, there was little influence on the survival capability of the biofilm cells (Figure 4B). Importantly, the results showed that the inhibition of phagocytosis was more beneficial for the survival of planktonic SS2 than the degradation of NETs, while NETs appear to play an important role in biofilm SS2 elimination.

### NETs bactericidal activity

**Figure 5:**
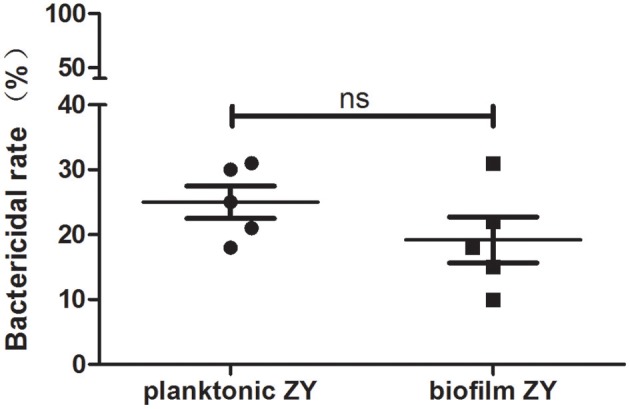
**Bactericidal capability of NETs**. The bactericidal rate was calculated by viable bacteria quantification on THB agar. The results are depicted as the mean ± SD (*n* = 5). ns, no difference between groups.

To evaluate the bactericidal capacity of NETs, the NETs bactericidal assay was performed. In the presence of NETs, nearly 25% of planktonic and 20% of biofilm SS2 cells were killed according to viable bacteria quantification on THB agar (Figure 5). The result indicated that NETs could kill both planktonic and biofilm SS2 and the bactericidal efficiency of NETs on planktonic SS2 and biofilm cells was comparable.

### Inhibition of NET formation by SS2 biofilm

**Figure 6:**
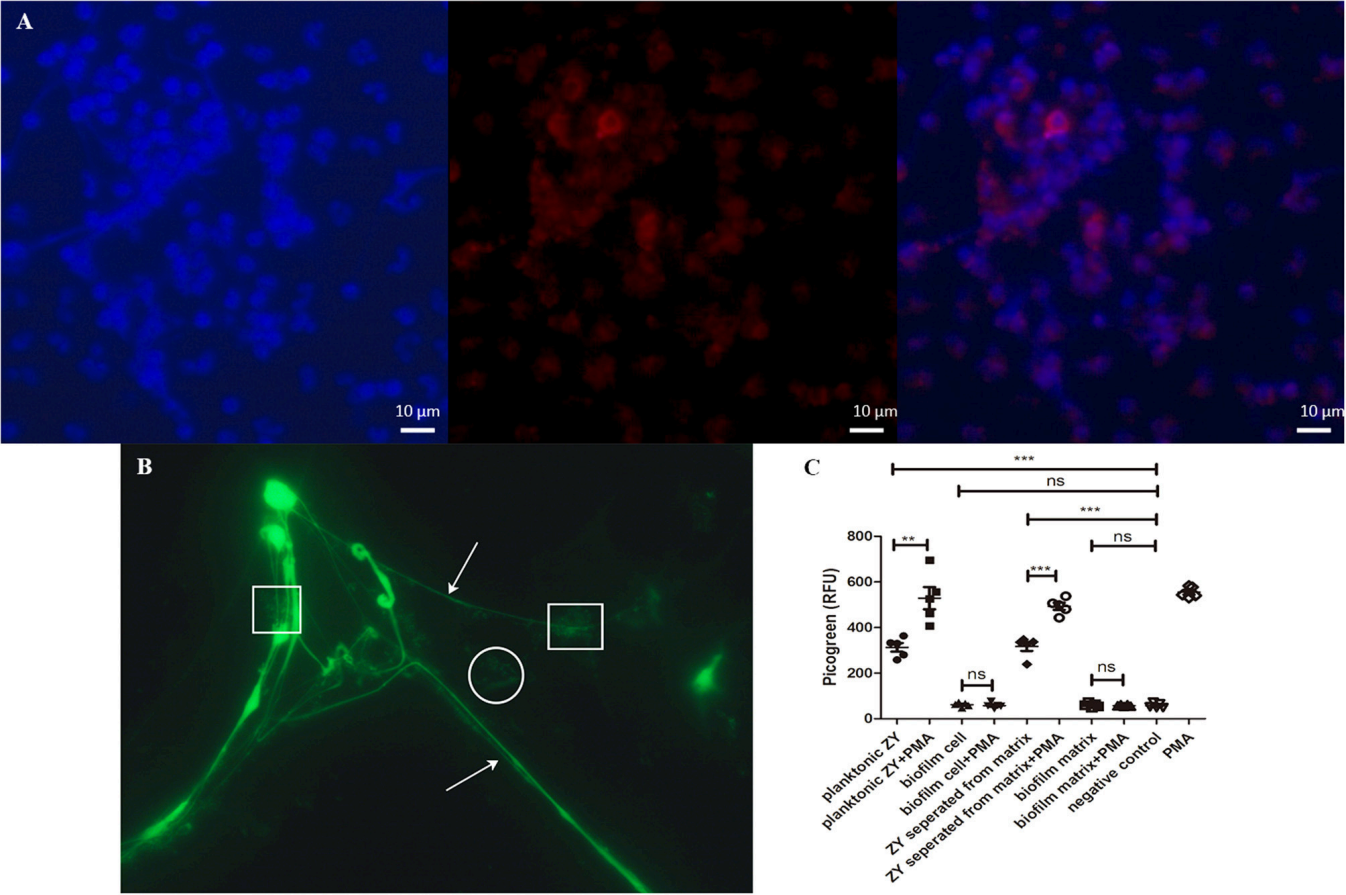
**NETs visualization and quantification. (A)** Neutrophils were stimulated with planktonic SS2. The pictures from the left to right were labeled with the following dyes: DNA with DAPI (blue), histone H4 (citrulline 3) with Alexa 568 conjugated (red), and an overlay of the first two pictures using ZEN 2012 software (Zeiss). Scale bar, 10 μm. **(B)** At 100 × magnification with oil, DNA was stained with the SYTO 9 green fluorescent nucleic stain. Arrows indicate the NETs structure; the round shapes indicate free bacteria without entrapment, and the square shapes indicate bacteria entrapped by NET DNA. **(C)** Relative fluorescence units were used to evaluate the quantity of NETs. Planktonic SS2 and bacteria separated from biofilm matrix could induce NETs release. The NET formation level induced by planktonic SS2 with PMA was twice that induced by planktonic SS2. The NET formation induced by bacteria separated from biofilm matrix with PMA-treated neutrophils was twice that induced by bacteria only. NETs induced by biofilm SS2 and the biofilm matrix were similar to the negative control. The results are depicted as the mean ± SD (*n* = 5). ^**^p < 0.01; ^***^p < 0.001; ns, no difference between groups.

Because NET formation is an important mechanism to kill SS2 biofilm, we next examined whether SS2 biofilms induced NET formation by neutrophils. SS2 ZY05719 could induce NETs release and could be captured by the NETs (Figures 6A,B).

To further study the influence of biofilms on NET formation, the NETs quantification assay was developed. Only planktonic SS2 and bacteria separated from biofilm extracellular matrix could stimulate NETs release compared to biofilm cells. Importantly, the supernatant of the dispersed biofilm mixture, which is mainly composed of biofilm extracellular matrix, could not stimulate NET formation. In this case, bacteria were incubated with PMA stimulated neutrophils to determine whether biofilms failed to activate neutrophils or inhibit NET formation. The extracellular DNA of NETs induced by planktonic SS2 and bacteria separated from biofilm matrix was enhanced by PMA; however the biofilms and biofilm extracellular matrix inhibited PMA-induced NETs as well (Figure 6C). These results indicated that bacteria both from the planktonic state and the dispersed biofilm state can stimulate NETs release; however, the extracellular biofilm matrix inhibited NET formation.

### Survival of planktonic and biofilm SS2 *in vivo*

**Figure 7:**
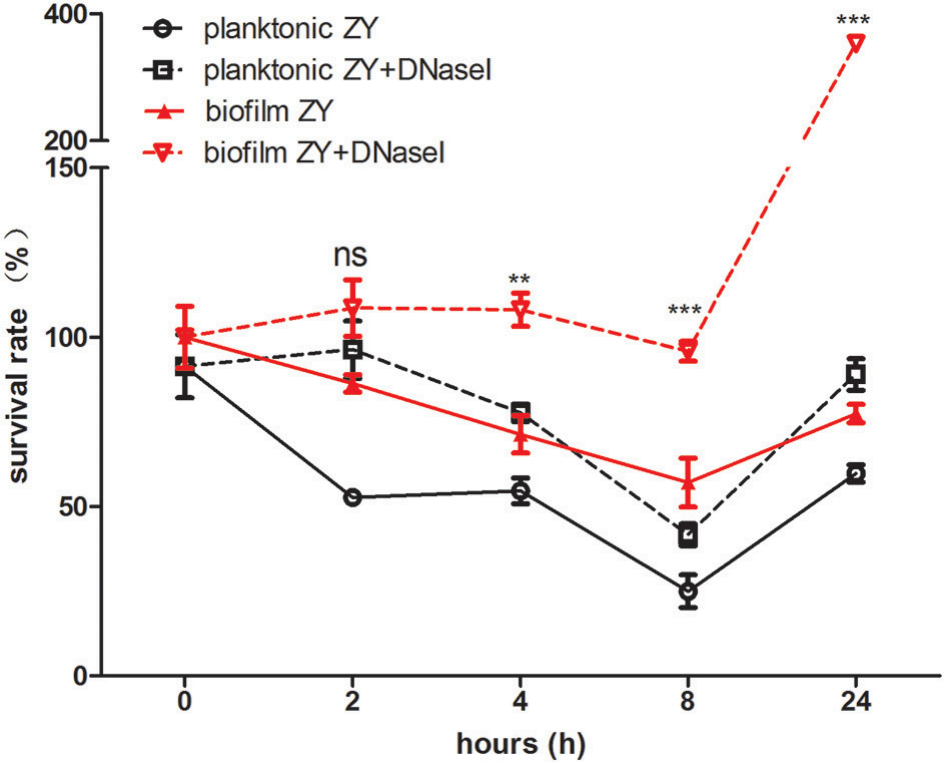
**Bacteria survival rate in mouse blood**. A comparison of the survival rate of bacteria isolated from mouse blood post-infection between planktonic and biofilm SS2 cells is shown. At 2, 4, 8, and 24 h post-infection, there were significantly more viable biofilm SS2 cells than planktonic SS2 cells. When DNase I was added to degrade NET DNA, the bacteria both in planktonic state and biofilm state displayed enhanced survival in the blood, particularly for biofilm SS2 cells. The survival rate between the group of planktonic ZY with DNase I and the group of biofilm ZY with DNase I was compared in each time point. The results are depicted as the mean ± SD (*n* = 3). ^**^p < 0.01; ^***^p < 0.001; ns, no difference between groups.

Following the observation that NETs kill both planktonic SS2 and biofilm SS2 *in vitro*, we aimed to identify the function of NETs in blood *in vivo*. In the first 8 h post-infection, both viable planktonic SS2 and viable biofilm SS2 cells decreased. This result may be ascribe to the host immune response. However, biofilm SS2 survived better than the planktonic cells in the blood stream. Notably, the survival of planktonic and biofilm SS2 was enhanced when NETs were degraded by DNase I. When NETs were destroyed with DNaseI, The viable bacteria in biofilm was much more than planktonic bacteria after 2 h post-infection (Figure 7). These results confirmed that biofilm SS2 demonstrated enhanced survival in the host, particularly when NET DNA was degraded.

## Discussion

SS2 is an important emerging zoonotic pathogen in humans (Smith et al., 2001; Goyette-Desjardins et al., 2014). Most of SS2 human invasive isolates formed biofilm according to previous studies (Bojarska et al., 2016). Bacterial biofilm formed on host surfaces, which is critical to the virulence of these organisms. Neutrophils initiate potent response to evasive pathogens and surveille the tissue in the circulation, which plays an essential role in innate immunity (Nicolas-Avila et al., 2017). These cells rapidly react to infection and clear pathogens. To date, little information is available on the interaction between neutrophil and SS2. It is critical to study the response of neutrophils to SS2. In this study, we examined the interaction between SS2 and neutrophils and SS2 biofilm formation was increased in the presence of neutrophils. SS2 biofilms can mediate neutrophil phagocytosis evasion; however, SS2 biofilms can be killed by NETs, and the bactericidal efficiency is comparable to the action of NETs on planktonic SS2. Importantly, SS2 biofilm cells can inhibit NET formation, mainly because of the biofilm extracellular matrix.

Bacterial biofilms can exist in a range of host tissues in the process of bacterial infection, which enables the bacterial communities to persist in the host (Boles and Horswill, 2011). From the perspective of the bacteria and host immune system relationship, pathogens form biofilms to increase chances of survival and to cause persistent infection in the host (Watters et al., 2016). For example, biofilm formation provides pneumococci with a protected environment for bacterial cells and enables transmission from person to person during nasopharyngeal colonization (Marks et al., 2013). Human neutrophils can enhance the development of *Pseudomonas aeruginosa* biofilms (Walker et al., 2005). In addition, previous studies have reported that macrophages and monocytes increase *C. albicans* biofilm formation (Chandra et al., 2007; Watters et al., 2016). In this study, the results showed that neutrophils can promote SS2 biofilm formation and that biofilm SS2 was better able to survive than planktonic cells in macrophages and neutrophils *in vitro* and in blood *in vivo*. To survive in the host, resistance to phagocytes in the blood is a crucial event for the pathogenicity of SS2 (Zhu et al., 2016). These findings suggest that biofilm formation is a survival strategy utilized by SS2 to evade phagocytosis. In addition, our results demonstrate that SS2 can form biofilms in some tissues such as the liver, spleen and kidney *in vivo*.

In addition to phagocytosis, neutrophils release NETs to trap and kill pathogens through extracellular DNA and antimicrobial proteins (Thammavongsa et al., 2013). Various pathogens can be killed by NETs including parasites, fungi, bacteria, and viruses (Saitoh et al., 2012; Uchiyama et al., 2015; Avila et al., 2016; Von Kockritz-Blickwede et al., 2016). It has previously been reported that a microbe size-sensing mechanism allows neutrophils to selectively respond to pathogens on the basis of microbe size. Small microbes are more likely to be taken up in a phagolysosome instead of stimulating NET formation (Branzk et al., 2014). One study showed that *C. albicans* with hyphae, which are too large to be phagocytized, are large enough to induce NET formation; however, *C. albicans* in yeast form failed to induce NETs release and *C. albicans* biofilms impaired NET formation (Branzk et al., 2014; Johnson et al., 2016). *C. albicans* biofilms consist of two main kinds of cells, small oval yeast-form cells and long tubular hyphal cells, and both yeast cells and hyphae are crucial for biofilm formation. SS2 and many pathogenic bacteria can induce NET formation. Therefore, virulence mechanisms may have a critical role in NET formation and microbe size may not the most important virulence mechanism that induce NETs in response to bacterial stimuli. SS2 biofilms are communities of bacteria with extracellular DNA, proteins and exopolysaccharides. Importantly, biofilm extracellular matrix can vary greatly depending on the microorganisms present. The different properties between bacterial pathogens and the fungal pathogen *C. albicans* may contribute to the different activities of biofilms in response to neutrophils. Importantly, phagocytosis is a much faster mechanism than NET formation and phagocytosis remains the major method for host immune cells to clear invasive SS2 cells (Fuchs et al., 2007; Nordenfelt and Tapper, 2011). A reasonable hypothesis is that NETs aid in the killing of SS2 biofilm cells that are difficult to phagocytose by immune cells and that NETs appear to be an important method of eliminating SS2 biofilm cells.

Both planktonic SS2 and biofilm SS2 cells can cause neutrophil accumulation at infection sites, providing an ideal environment for NETs immunoreaction. Significantly, NETs appear to have equal bactericidal efficacy for biofilm and planktonic SS2 cells. Neutrophils were treated with DNase I and cytochalasin B to degrade NETs and to suppress phagocytosis, respectively. For planktonic SS2, when NETs and phagocytosis were suppressed separately, the bacterial survival in neutrophils was improved significantly. These results indicated that NET formation and phagocytosis are both important mechanisms for killing invasive planktonic SS2, which is consistent with previous reports (De Buhr et al., 2017). Planktonic bacteria were more likely to be cleared by phagocytosis. When NET DNA was degraded, the survival of SS2 biofilm cells increased; however, phagocytosis had no obvious bactericidal effect on biofilm bacteria. In addition, in blood survival assays *in vivo*, biofilm cells were better able to survive compared to planktonic cells. When NET DNA was degraded, biofilms protected the bacteria from being killed and biofilm cells had enhanced survival *in vivo*, indicating that NETs could be an important bactericidal mechanism to entrap and kill bacteria biofilms in the host blood stream. Both phagocytosis and NETs are important bactericidal mechanisms for planktonic cells, and planktonic SS2 can stimulate NETs release and can be entrapped by NETs. NET formation appeared to be an efficient bactericidal mechanism for biofilm cells in this study; however, bacterial biofilms and the biofilm extracellular matrix could inhibit NET formation even in the presence of PMA, indicating that biofilms inhibit NETs release mainly through the extracellular matrix. Notably, bacteria separated from biofilms matrix still have the ability to induce NET formation. Further work will address on the mechanism of NET inhibition through biofilm matrix.

Secretion of nuclease has been the main strategy to degrade the NET DNA backbone for bacteria in previous studies (Uchiyama et al., 2012). In this study we found that biofilm is another mechanism to inhibit NETs release. Importantly, SS2 biofilms inhibit NETs release through the biofilm extracellular matrix. Biofilms are significant protective shelters for bacteria and enable survival by allowing the pathogen to persist and resist the host immune system. Although biofilms can evade phagocytosis and inhibit NET formation, NETs derived from neutrophils stimulated by planktonic bacteria and host inflammatory factors might be a significant mechanism of eliminating bacterial biofilms. This study provides novel knowledge on the battles between NETs and bacterial biofilms and can potentially inform novel strategies for the clinical treatment of streptococcal disease.

## Author contributions

All authors have significant contributions to the completion of the manuscript. Conception and design of the work: FM and HF; acquisition and analysis the data: FM, NY, and GW; interpretation of data for the work: FM, LY, NY, and HF; drafting and revising the work: FM, ZM, HL, and HF; final approval and agreement to be accountable for all aspects of the work: FM, LY, NY, GW, ZM, HL, and HF.

## Funding

This study was supported by grants from the National Transgenic Major Program (2014ZX0800946B), the Special Fund for Agro-scientific Research in the Public Interest (201403054), the National Natural Science Foundation of China (31272581, 31672574), the Jiangsu Agriculture Science and Technology Innovation Fund (CX(16)1028), and the Priority Academic Program Development of Jiangsu Higher Education Institutions (PAPD).

### Conflict of interest statement

The authors declare that the research was conducted in the absence of any commercial or financial relationships that could be construed as a potential conflict of interest.
